# Assessment of the Route of Exposure to Ovalbumin and Cow’s Milk Proteins on the Induction of IgE Responses in BALB/c Mice

**DOI:** 10.3390/biology11040542

**Published:** 2022-03-31

**Authors:** Feliznando Isidro Cárdenas-Torres, Francisco Cabrera-Chávez, Aldo Alejandro Arvizu-Flores, Lilian Karem Flores-Mendoza, Veronica Lopez-Teros, Humberto Astiazaran-Garcia, Martina Hilda Gracia-Valenzuela, Oscar Gerardo Figueroa-Salcido, Jesús Gilberto Arámburo-Gálvez, Noé Ontiveros

**Affiliations:** 1 Faculty of Nutrition Sciences, Nutrition Sciences Postgraduate Program, University of Sinaloa, Culiacán 80019, Sinaloa, Mexico; feliznando@uas.edu.mx (F.I.C.-T.); fcabrera@uas.edu.mx (F.C.-C.); oscar.figueroa@uas.edu.mx (O.G.F.-S.); 2 Division of Biological and Health Sciences, Postgraduate Program in Health Sciences, University of Sonora, Hermosillo 83000, Sonora, Mexico; aldo.arvizu@unison.mx (A.A.A.-F.); veronica.lopez@unison.mx (V.L.-T.); 3 Division of Sciences and Engineering, Department of Chemical, Biological, and Agricultural Sciences (DC-QB), Clinical and Research Laboratory (LACIUS, URS), University of Sonora, Navojoa 85880, Mexico; lilian.flores@unison.mx; 4 Department of Nutrition, Research Center for Food and Development, CIAD, A.C., Hermosillo 83304, Mexico; hastiazaran@ciad.mx; 5 National Technological of Mexico/Technological Institute of the Yaqui Valley, Bácum 85276, Mexico; mgracia.valenzuela@itvy.edu.mx

**Keywords:** food allergy, intragastric sensitization, intraperitoneal sensitization, BALB/c mice, ovalbumin, cow’s milk proteins

## Abstract

**Simple Summary:**

The mouse model is very valuable to scientists, mainly because many reagents are commercially available for conducting research in this animal model. In the search for a standardized mouse model of food allergy, the route of administration of the proteins of interest is a determinant to successfully sensitizing the mice. Our aim was to evaluate the IgE allergen-specific response to food proteins after their administration by the intragastric or intraperitoneal routes in BALB/c mice. The results show that the intraperitoneal administration of the allergens ovalbumin or cow’s milk protein triggered more robust and consistent immunoglobulin E responses than the intragastric administration of the proteins, whether Sucralfate is used or not (an antacid that can promote sensitization in mice and an allergic response similar to the one triggered in human beings). It is concluded that the intraperitoneal administration of food proteins is better than the intragastric one to sensitize BALB/c mice, even after gastric-acid suppression. We have generated scientific evidence to pave the way in the search for a reproducible mouse model of immunoglobulin E-mediated food allergy to evaluate the safety of crops derived from modern biotechnology, such as genetic engineering, or the safety and effectiveness of new food allergy therapies.

**Abstract:**

BALB/c mice can be orally sensitized to food proteins under acid suppressive medication, mimicking human exposure and triggering a human-like allergic immune response. However, the reproducibility of such an oral food allergy model remains questionable. Our aim was to evaluate the IgE responses triggered against ovalbumin (OVA) and cow’s milk proteins (CMP) after intragastric (IG), either under gastric-acid suppression or not, or intraperitoneal (IP) sensitization in BALB/c mice. OVA (0.2 mg) and different concentrations of CMP were administered with/without the antacid sucralfate by the IG route. For IP sensitization, OVA or CMP (0.5 mg) were administered. ELISA was used to evaluate IgE responses. The IP sensitization protocols triggered more robust and consistent anti-OVA or anti-CMP IgE responses than the intragastric ones (with/without sucralfate) (*p* < 0.05). 2.7% (1/36), and 5.5% (3/54) of the mice that underwent the sucralfate-assisted IG protocol triggered IgE responses against OVA or CMP, respectively. All the mice were administered OVA or CMP via IP triggered detectable IgE responses. The IP sensitization model is more reliable than the IG one for evaluating the intrinsic sensitizing and/or allergenic potential of food proteins, even if IG immunizations are carried out under gastric-acid suppression.

## 1. Introduction

IgE-mediated food allergy (FA) is an abnormal immune response triggered in susceptible individuals after the ingestion of allergenic food proteins [1]. This disorder affects around 8% of children and 4% of the general population [2,3,4]. Although a variety of potential FA therapies have been proposed [5], the only accepted treatment for the condition is to avoid the allergen of interest. Both new FA therapies and foods produced through modern biotechnology ideally should undergo safety assessments in animal models of FA before their evaluation in humans or their introduction into the human diet, respectively [6]. Animal models of FA have enormous potential as a research tool and, consequently, some models have been developed (e.g. sheep, dog, swine, mouse, etc.) [7]. The mouse model of FA has gained popularity over other models mainly due to the commercial availability of many mouse-specific immunological reagents, their short generation time, the possibility of large experimental groups of animals, and the relatively low cost of purchase and maintenance [8]. In particular, the BALB/c mouse strain has been widely used for evaluating the sensitizing or allergenic potential of proteins [9,10,11,12,13,14,15,16,17,18,19] because this strain tends to trigger Th2 immune responses with atopic-like phenotype [16,17]. However, to be successful in sensitizing BALB/c mice while saving time and resources, the sensitization route should be carefully chosen.

In mice, the most common sensitization routes are the intragastric and the intraperitoneal (IP) administration of proteins. The oral sensitization route is expected to occur in most FA cases and, therefore, it seems to possess a sizeable advantage over the IP administration of proteins. The main limitation of the oral sensitization route is the fact that the mucosal immune system naturally triggers tolerogenic immune responses instead of other types of lymphocyte-mediated immunity, e.g., IgE-mediated responses [20,21,22]. Adjuvants can be used to overcome the mucosal immune tolerance to food proteins in mice. However, an adjuvant-free sensitization protocol should be chosen for the evaluation of the inherent sensitizing or allergenic potential of proteins or the assessment of the effectiveness of new FA therapies to minimize false negative results. Almost two decades ago, it was highlighted that the IP route performs better than the intragastric one to sensitize BALB/c mice [23], but others have highlighted that the use of antacids can help to sensitize the BALB/c strain through the intragastric route [24,25]. The use of the antacid Sucralfate, which contains aluminum and is recommended for the treatment of gastric ulcers, has been claimed to promote sensitization to food proteins in mice and to trigger allergic responses similar to those triggered in human beings [24]. An orally sensitized model of FA that triggers an allergic immune response that resembles the one triggered in food allergic individuals would be a very important step forward in the search for a standardized animal model of FA if reproducible results can be obtained. Thus, due to the scarcity of information about the allergen-dependent reproducibility of the Sucralfate-assisted BALB/c mouse model of FA, our aim was to evaluate in BALB/c mice the IgE allergen-specific response after intragastric, either Sucralfate-assisted or not, or intraperitoneal sensitization using the reference allergen ovalbumin (OVA) and cow’s milk protein, the latter being a well-known allergen that hardly sensitizes mice under adjuvant-free conditions.

## 2. Materials and Methods

### 2.1. Allergens and Protein Quantification

Ovalbumin (OVA; grade V, ≥98% purity) and cow’s milk protein (CMP) rich in casein (87.1% [10]) were obtained from Sigma Chemical (Sigma-Aldrich, Saint Louis, MO, USA) and MP Biomedicals (Oklahoma, OH, USA), respectively. The test proteins were dissolved in PBS pH 7.4 (Sigma-Aldrich, Saint Louis, MO, USA; Cat: P3813), and the protein concentrations were determined (BCA assay, Thermo Scientific, Rockford, IL, USA).

### 2.2. Animals and Ethical Aspects

A total of 136 five- to six-week-old female BALB/c mice were used in this study (Bioterium Claude Bernard, Benemerita Universidad Autónoma de Puebla, Puebla, México). The mice were free of pathogens and randomly assigned to one group or another. The diet of the mice was free of cow’s milk and egg-protein, and the animals were fed with this diet for three generations (Mazuri—Rat & Mouse Diet #5663). Water and diet were available ad libitum. The mice were housed in an animal room under standard conditions [9]. The ethics review board of the Autonomous University of Sinaloa (Universidad Autónoma de Sinaloa) approved the study protocol (Ethical approval number: CE-UACNyG-2014-JUL-001).

### 2.3. Intragastric and Intraperitoneal (IP) Sensitization

For intragastric (IG) sensitization, groups of 5 to 36 mice were administered 0.2 mg of OVA or different doses (0.2, 0.4, 1.0, 2.5 and 4.0 mg) of CMP in a final volume of 150 µL using feeding tubes (Instech Laboratories, Inc, Pennsylvania, United States, cat. FTP-20-30). The Sucralfate groups received the protein of interest plus 50 µL of Sucralfate (1 g/5 mL suspension: Unival, Senosiain) in a final volume of 150 µL. This procedure was carried out on days 0, 7, 14, 21, 28, 35, 42 and 49 [19] (Table 1). The control group received 150 µL of PBS only. Blood samples were collected from the tail vein on days 0 and 52. For IP purposes, the mice were sensitized as previously described [9,10]. Briefly, the mice received two different protocols of IP sensitization 250 μL of 0.02% OVA (0.05 mg) or 0.05 mg of CMP in a final volume of 250 μL (Table 1). For the 28-day protocol, the treatments were repeated on days 3, 6, 9 and 12 after the first administration of the proteins (day 0). For the 35-day protocol, the treatments were repeated on days 14 and 28. The control group received 250 μL of PBS only. Blood samples were collected from the tail vein on days 28 or 35 (Table 1). As a positive control, groups of mice were sensitized IP using an aqueous solution containing aluminum hydroxide and magnesium hydroxide (40 mg/mL, each) (Imject Alum; Thermo Scientific; Cat: 77161). For this purpose, 50 µg of the protein of interest and 2 mg of aluminum hydroxide were administered in 100 μL. The procedure was repeated on day 14 after the first protein administration (day 0), and blood samples were collected on day 16 (Table 1).

### 2.4. IgE Responses

Anti-OVA or -CMP IgE were detected using ELISA. Briefly, antigens (20 μg) in coating buffer were added to each well (96-well plates) and incubated overnight at 4 °C. The wells were blocked, and serum samples (100 μL) diluted to 1:10 or 1:50 (Imject Alum groups) added to the wells. After overnight incubation at 4 °C, the wells were washed. Detection antibody and streptavidin-horseradish peroxidase were obtained from BioLegend Inc, San Diego, California, United States. Tetramethyl benzidine and H_2_SO_4_ 2 M were used as a substrate and a stop solution, respectively. All serum samples were evaluated in triplicate and measurements carried out at 450 nm. The ELISA assay was previously described in detail [9]. The results are presented as an absorbance Fold-change, determined as follows:

(1) $$\mathrm{Fold}\;\mathrm{change}=\frac{\left(\mathrm{absorbance}\;\mathrm{from}\;\mathrm{immnue}\;\mathrm{serum}-\mathrm{absorbance}\;\mathrm{from}\;\mathrm{preimmune}\;\mathrm{serum}\right)}{\mathrm{absorbance}\;\mathrm{from}\;\mathrm{preimmune}\;\mathrm{serum}}$$

A 4-fold-change in absorbance at 450 nm was considered as an increased IgE response (optical density values can be found as Supplementary material (Table S1)).

### 2.5. Statistical Analysis

Data were analyzed using GraphPad Prism Version 5.0 (GraphPad Software, San Diego, CA, USA). A Shapiro-Wilk test was applied to evaluate data normality. A Brown-Forsythe and a Weal ANOVA test followed by an unpaired *t*-test with Welch’s correction were used for comparisons among IgE responses in the experimental groups. A *p* value < 0.05 was considered statistically significant.

## 3. Results

### 3.1. The IG Administration of OVA with or without Sucralfate Is Less Effective than the IP Route to Sensitize BALB/c Mice

**Figure 1:**
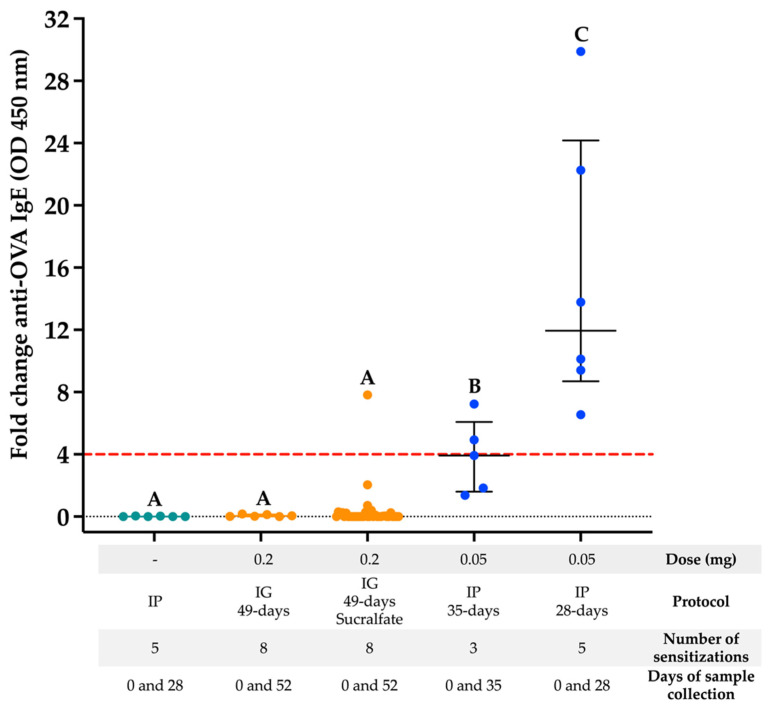
Comparison between the intragastric and IP routes of sensitization to OVA in BALB/c mice. The presence of anti-OVA IgE was evaluated using ELISA. Different letters represent a statistically significant difference (*p* < 0.05). IG: intragastric; IP: intraperitoneal; OVA: ovalbumin. Green dots: control group; Orange dots: IG protocol (*n* = 6 or 36); Blue dots: IP protocol (*n* = 5 or 6); Dotted line: 4-Fold change cut-off value.

The sensitization protocols evaluated, either intragastric (one protocol) or intraperitoneal (two protocols), triggered anti-OVA IgE immune responses that were detected using ELISA (Figure 1). Regarding the intragastric protocol, only 1 out of 36 mice (2.7%) that underwent the Sucralfate-assisted protocol of sensitization showed detectable anti-OVA IgE responses above the arbitrary cut-off (4-fold-change). The 28- and 35-day intraperitoneal protocols of sensitization triggered more robust and consistent anti-OVA IgE responses than the intragastric one (*p* < 0.05) (Figure 1). The 28-day IP protocol triggered a higher anti-OVA IgE response than the 35-day IP protocol (*p* < 0.05).

### 3.2. The IG Administration of CMP with or without Sucralfate Is Less Effective than the IP Route to Sensitize BALB/c Mice

**Figure 2:**
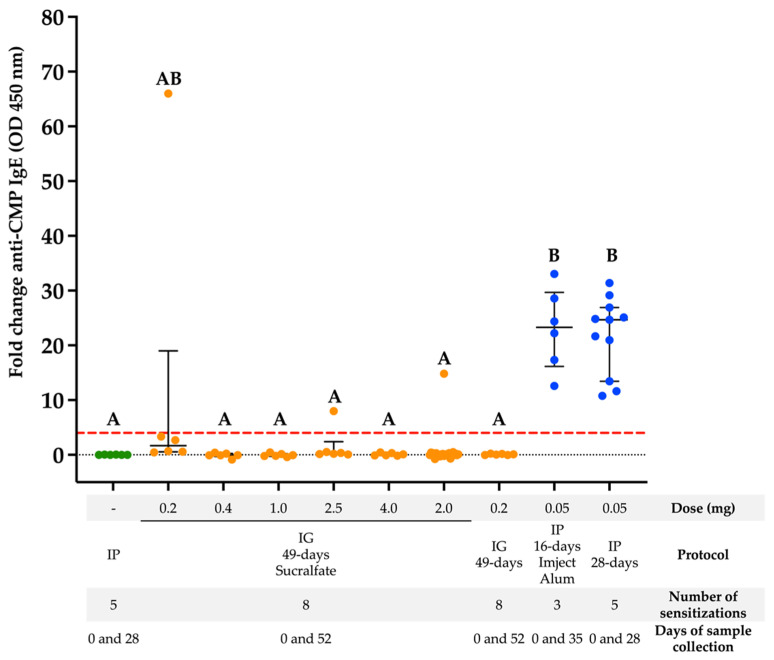
Comparison between the intragastric and IP routes of sensitization to CMP in BALB/c mice. The presence of anti-CMP IgE was evaluated using ELISA. Different letters represent a statistically significant difference (*p* < 0.05). IG: intragastric; IP: intraperitoneal; CMP: cow’s milk proteins. Green dots: control group; Orange dots: IG protocol (*n* = 6 or 24); Blue dots: IP protocol (*n* = 6 or 11); Dotted line: 4-Fold change cut-off value.

Groups of 6 mice underwent the Sucralfate-assisted protocol of sensitization using different doses of CMP. The group that received 0.2 mg of CMP by the IG route showed a better IgE response than the other groups (Figure 2). Consequently, a second group of mice underwent the Sucralfate-assisted protocol using the dose of 0.2 mg of CMP (*n* = 24). However, only 1 out of 24 mice (4.1%) triggered detectable IgE responses above the arbitrary cut-off (4-fold-change). Contrary, all mice that underwent the 28-day IP protocol, either with or without the use of adjuvant, triggered anti-CMP IgE responses readily detected using ELISA (Figure 2).

## 4. Discussion

Female BALB/c mice were used to evaluate intragastric and IP sensitization to cow’s milk and egg proteins. Certainly, IP sensitization avoids both gastrointestinal digestion and the tolerogenic immune response triggered against food proteins in the gut-associated lymphoid tissue, making this sensitization route more effective than the IG one. However, findings highlight that the use of the antacid Sucralfate can serve as an adjunct to sensitize up to 85% of the mice treated [26] and that the allergic response elicited is similar to the one triggered in human beings [24]. In the present study, an IP 28-day sensitization protocol [12] and a 35-day one were used. The intragastric sensitization was evaluated using a 49-day protocol, either with or without the use of the antacid sucralfate [19], which is an aluminum-sucrose-sulfate complex used for the treatment of gastric ulcers [25]. Sucralfate increases the stomach pH [19], reducing the activity of the digestive enzyme pepsin, and it is a source of aluminum hydroxide, which promotes Th2-type responses and the production of IgE [27,28]. Under these bases, the egg-protein sensitization of BALB/c mice has been widely documented after gastric acid-suppression with Sucralfate [19]. However, the lack of sensitization in some BALB/c mice using gastric acid-suppression with Sucralfate was documented and attributed to changes in intestinal bacterial colonization patterns between the sensitized and non-sensitized mice [26]. As stated before, this could be of minor relevance in some research laboratories, since the authors stated that almost 85% of the mice were sensitized [26]. Contrary to this, only 2.77% (1 out 36) of the mice that underwent the Sucralfate-assisted intragastric protocol of sensitization triggered anti-OVA IgE responses readily detected using ELISA in the present study. Unfortunately, we were unable to evaluate the bacterial colonization patterns of the mice, but we believe that if this is a practice for choosing which animals can be sensitized and which cannot, the Sucralfate-assisted intragastric protocol can hardly be considered simple and suitable for many research laboratories. Notably, the IP sensitization to OVA was successfully performed in all the groups of mice, either using adjuvant or not, and, on average, the IgE immune responses were more robust and consistent than those triggered by the mice that underwent the Sucralfate-assisted protocol. Therefore, the results support the notion that the IP sensitization to OVA performs better than the intragastric one, as well as highlighting that a large number of BALB/c mice should undergo the Sucralfate-assisted intragastric protocol of mice sensitized via the oral route.

Some reported that BALB/c mice do not become sensitized to CMP through the intragastric route, even using cholera toxin as adjuvant [29]. The present study does not support such a statement, since 3 mice that underwent the Sucralfate-assisted sensitization protocol triggered anti-CMP IgE immune responses readily detected using ELISA. In fact, excluding the group of mice sensitized with the use of Imject Alum, the most robust anti-CMP IgE immune responses were triggered after the intragastric administration of 0.2 mg of CMP and Sucralfate. It should be noted that the BALB/c mouse strain could be genetically not as susceptible as the C3H/HeJ one to the development of a cow’s milk allergy [30], but it remains unknown if the C3H/HeJ mouse strain depends on the use of cholera toxin to be sensitized to CMP. If cholera toxin is needed, the C3H/HeJ mouse strain would not be appropriated to evaluate the intrinsic allergenic or sensitizing potential of CMP. Overall, in the present study, only 5 out of 54 mice (9.25%) triggered anti-CMP IgE immune responses detected using ELISA, but only 3 of these 5 mice (5.5%) showed robust anti-CMP IgE responses. As stated by others [26], the bacterial colonization patterns of the mice could be relevant for skewing the potential allergenic immune response to food proteins to a tolerogenic one.

The lack of sensitization to CMP can be avoided by sensitizing the BALB/c mice through the IP route. In line with previous reports [10], the present study shows that the IP administration of 0.05 mg of CMP rich in caseins can efficiently sensitize BALB/c mice in an adjuvant-free manner. In fact, all mice that underwent the 28-day adjuvant-free IP protocol triggered anti-CMP IgE responses readily detected using ELISA. These results highlight that the Sucralfate-assisted sensitization protocol is less efficient in sensitizing BALB/c mice to CMP than the IP one. Certainly, mice have a short generation time, and they are relatively low-cost in terms of purchase and maintenance, but the proportion of BALB/c mice sensitized to CMP or OVA through the intragastric route is quite low, and this fact could complicate the use of the Sucralfate-assisted sensitization protocol in studies that require dozens of sensitized BALB/c mice.

## 5. Conclusions

The present study confirms that the route of food proteins administration is decisive for successfully sensitizing BALB/c mice and that the IP route is more efficient than the intragastric one for triggering IgE responses against food proteins in this mouse strain, even if immunizations with gastric-acid suppression with sucralfate are carried out.

## Figures and Tables

**Table 1:** Intragastric and IP protocols of sensitization.

Allergen	Dose (mg)	Sensitization Route	Adjuvant	Frequency of Sensitization	Number of Sensitizations	Blood Samples (Days)
Ovalbumin	0.05	IP	None	Every 3 days	5 (days 0, 3, 6, 9 and 12)	0 and 28
	0.05	IP	None	Every 14 days	3 (days 0, 14 and 28)	0 and 35
	0.2	IG	Sucralfate	Weekly	8 (days 0, 7, 14, 21, 28, 35, 42 and 49)	0 and 52
Cow’s Milk Proteins	0.05	IP	None	Every 3 days	5 (days 0, 3, 6, 9 and 12)	0 and 28
	0.05	IP	Imject Alum	Every 14 days	2 (days 0 and 14)	0 and 16
	0.2 0.4 1.0 2.5 4.0	IG	Sucralfate	Weekly	8 (days 0, 7, 14, 21, 28, 35, 42 and 49)	0 and 52

IP: Intraperitoneal; IG: Intragastric.

## Data Availability

All data are available within the article and supplementary material.

## Supplementary Materials

The following supporting information can be downloaded at: https://www.mdpi.com/article/10.3390/biology11040542/s1, Table S1 Optical density values of IgE responses.
