# Supplementary material for: Assessment of the Route of Exposure to Ovalbumin and Cow’s Milk Proteins on the Induction of IgE Responses in BALB/c Mice
Source: Biology (Basel). 2022 Mar 31;11(4):542. doi: 10.3390/biology11040542 (PMC9031655; doi:10.3390/biology11040542)
Supplement: Supplementary file 1 [file biology-11-00542-s001.zip › biology-1614034-supplementary.pdf]

Supplementary material

Table S1: Optical density values of IgE responses

| Doses and Allergen | Protocol of Sensitization | Nember of Sensitization | Days of Sample Collection | Mice | OD Values 450 nm | | Fold-Change |
|---|---|---|---|---|---|---|---|
| | | | | | Preimmnune | Preimmnune | |
| 0.2 mg CMP | IG 49-days | 8 | 0 and 52 | 1 | 0.015 | 0.016 | 0.067 |
| | | | | 2 | 0.015 | 0.014 | -0.067 |
| | | | | 3 | 0.018 | 0.022 | 0.222 |
| | | | | 4 | 0.018 | 0.020 | 0.111 |
| | | | | 5 | 0.019 | 0.018 | -0.053 |
| | | | | 6 | 0.017 | 0.020 | 0.176 |
| 0.2 mg CMP | IG 49-days Sucralfate | 8 | 0 and 52 | 1 | 0.014 | 0.944 | 66.429 |
| | | | | 2 | 0.011 | 0.048 | 3.364 |
| | | | | 3 | 0.012 | 0.044 | 2.667 |
| | | | | 4 | 0.010 | 0.014 | 0.400 |
| | | | | 5 | 0.008 | 0.013 | 0.625 |
| | | | | 6 | 0.011 | 0.018 | 0.636 |
| 0.4 mg CMP | IG 49-days Sucralfate | 8 | 0 and 52 | 1 | 0.020 | 0.019 | -0.050 |
| | | | | 2 | 0.012 | 0.010 | -0.167 |
| | | | | 3 | 0.014 | 0.016 | 0.143 |
| | | | | 4 | 0.014 | 0.020 | 0.429 |
| | | | | 5 | 0.009 | 0.005 | -0.444 |
| | | | | 6 | 0.011 | 0.009 | -0.182 |
| 1.0 mg CMP | IG 49-days Sucralfate | 8 | 0 and 52 | 1 | 0.015 | 0.021 | 0.400 |
| | | | | 2 | 0.014 | 0.013 | -0.071 |
| | | | | 3 | 0.012 | 0.014 | 0.167 |
| | | | | 4 | 0.010 | 0.009 | -0.100 |
| | | | | 5 | 0.093 | 0.013 | -0.860 |
| | | | | 6 | 0.022 | 0.020 | -0.091 |
| 2.5 mg CMP | IG 49-days Sucralfate | 8 | 0 and 52 | 1 | 0.099 | 0.105 | 0.061 |
| | | | | 2 | 0.027 | 0.031 | 0.148 |

| | | | | | | | |
|---|---|---|---|---|---|---|---|
| | | | | 3 | 0.017 | 0.020 | 0.176 |
| | | | | 4 | 0.019 | 0.171 | 8.000 |
| | | | | 5 | 0.009 | 0.014 | 0.556 |
| | | | | 6 | 0.015 | 0.020 | 0.333 |
| 4.0 mg CMP | IG 49-days Sucralfate | 8 | 0 and 52 | 1 | 0.023 | 0.019 | -0.174 |
| | | | | 2 | 0.022 | 0.024 | 0.091 |
| | | | | 3 | 0.017 | 0.015 | -0.118 |
| | | | | 4 | 0.010 | 0.009 | -0.100 |
| | | | | 5 | 0.009 | 0.013 | 0.444 |
| | | | | 6 | 0.013 | 0.017 | 0.308 |
| 0.2 mg CMP (Repetition) | IG 49-days Sucralfate | 8 | 0 and 52 | 1 | 0.083 | 0.108 | 0.301 |
| | | | | 2 | 0.072 | 0.081 | 0.125 |
| | | | | 3 | 0.073 | 0.095 | 0.301 |
| | | | | 4 | 0.084 | 0.078 | -0.071 |
| | | | | 5 | 0.067 | 0.073 | 0.090 |
| | | | | 6 | 0.064 | 0.067 | 0.047 |
| | | | | 7 | 0.065 | 0.079 | 0.215 |
| | | | | 8 | 0.067 | 0.095 | 0.418 |
| | | | | 9 | 0.082 | 0.081 | -0.012 |
| | | | | 10 | 0.066 | 0.072 | 0.091 |
| | | | | 11 | 0.067 | 0.078 | 0.164 |
| | | | | 12 | 0.081 | 0.073 | -0.099 |
| | | | | 13 | 0.06 | 0.089 | 0.483 |
| | | | | 14 | 0.234 | 0.075 | -0.679 |
| | | | | 15 | 0.051 | 0.067 | 0.314 |
| | | | | 16 | 0.239 | 0.056 | -0.766 |
| | | | | 17 | 0.072 | 0.069 | -0.042 |
| | | | | 18 | 0.073 | 1.155 | 14.822 |
| | | | | 19 | 0.06 | 0.067 | 0.117 |
| | | | | 20 | 0.071 | 0.056 | -0.211 |
| | | | | 21 | 0.058 | 0.07 | 0.207 |
| | | | | 22 | 0.078 | 0.076 | -0.026 |

| | | | | | | | |
|---|---|---|---|---|---|---|---|
| | | | | 23 | 0.077 | 0.055 | -0.286 |
| | | | | 24 | 0.071 | 0.055 | -0.225 |
| 0.05 mg CMP | IP 16-days Imject Alum | 2 | 0 and 14 | 1 | 0.010 | 0.296 | 28.600 |
| | | | | 2 | 0.013 | 0.330 | 24.385 |
| | | | | 3 | 0.011 | 0.374 | 33.000 |
| | | | | 4 | 0.009 | 0.209 | 22.222 |
| | | | | 5 | 0.018 | 0.244 | 12.556 |
| | | | | 6 | 0.016 | 0.293 | 17.313 |
| 0.05 mg CMP | IP 28-days | 5 | 0 and 35 | 1 | 0.008 | 0.259 | 31.375 |
| | | | | 2 | 0.009 | 0.198 | 21.000 |
| | | | | 3 | 0.019 | 0.491 | 24.842 |
| | | | | 4 | 0.007 | 0.180 | 24.714 |
| | | | | 5 | 0.012 | 0.272 | 21.667 |
| | | | | 6 | 0.014 | 0.365 | 25.071 |
| | | | | 7 | 0.011 | 0.139 | 11.636 |
| | | | | 8 | 0.017 | 0.245 | 13.412 |
| | | | | 9 | 0.009 | 0.251 | 26.889 |
| | | | | 10 | 0.018 | 0.212 | 10.778 |
| | | | | 11 | 0.010 | 0.301 | 29.100 |
| 0.05 mg OVA | IG 49-days | 8 | 0 and 52 | 1 | 0.078 | 0.082 | 0.051 |
| | | | | 2 | 0.071 | 0.080 | 0.127 |
| | | | | 3 | 0.072 | 0.084 | 0.167 |
| | | | | 4 | 0.080 | 0.081 | 0.013 |
| | | | | 5 | 0.075 | 0.074 | -0.013 |
| | | | | 6 | 0.083 | 0.085 | 0.024 |
| 0.05 mg OVA | IG 49-days Sucralfate | 8 | 0 and 52 | 1 | 0.086 | 0.08 | -0.070 |
| | | | | 2 | 0.095 | 0.113 | 0.189 |
| | | | | 3 | 0.379 | 0.531 | 0.401 |
| | | | | 4 | 0.097 | 0.085 | -0.124 |
| | | | | 5 | 0.1 | 0.11 | 0.100 |
| | | | | 6 | 0.083 | 0.074 | -0.108 |
| | | | | 7 | 0.559 | 0.696 | 0.245 |

| | | | | | | | |
|---|---|---|---|---|---|---|---|
| | | | | 8 | 0.11 | 0.078 | -0.291 |
| | | | | 9 | 0.482 | 0.606 | 0.257 |
| | | | | 10 | 0.648 | 0.48 | -0.259 |
| | | | | 11 | 0.104 | 0.316 | 2.038 |
| | | | | 12 | 0.494 | 0.645 | 0.306 |
| | | | | 13 | 0.64 | 0.549 | -0.142 |
| | | | | 14 | 0.634 | 0.523 | -0.175 |
| | | | | 15 | 0.082 | 0.068 | -0.171 |
| | | | | 16 | 0.101 | 0.082 | -0.188 |
| | | | | 17 | 0.491 | 0.631 | 0.285 |
| | | | | 18 | 0.097 | 0.855 | 7.814 |
| | | | | 19 | 0.59 | 0.608 | 0.031 |
| | | | | 20 | 0.087 | 0.076 | -0.126 |
| | | | | 21 | 0.706 | 0.498 | -0.295 |
| | | | | 22 | 0.817 | 0.679 | -0.169 |
| | | | | 23 | 0.703 | 0.397 | -0.435 |
| | | | | 24 | 0.897 | 0.528 | -0.411 |
| | | | | 25 | 0.095 | 0.083 | -0.126 |
| | | | | 26 | 0.092 | 0.091 | -0.011 |
| | | | | 27 | 0.318 | 0.389 | 0.223 |
| | | | | 28 | 0.104 | 0.086 | -0.173 |
| | | | | 29 | 0.095 | 0.074 | -0.221 |
| | | | | 30 | 0.386 | 0.662 | 0.715 |
| | | | | 31 | 0.408 | 0.326 | -0.201 |
| | | | | 32 | 0.085 | 0.066 | -0.224 |
| | | | | 33 | 0.088 | 0.081 | -0.080 |
| | | | | 34 | 0.091 | 0.075 | -0.176 |
| | | | | 35 | 0.558 | 0.58 | 0.039 |
| | | | | 36 | 0.683 | 0.456 | -0.332 |
| 0.05 mg OVA | IP 35-day | 3 | 0 and 35 | 1 | 0.082 | 0.675 | 7.232 |
| | | | | 2 | 0.087 | 0.428 | 3.920 |
| | | | | 3 | 0.095 | 0.225 | 1.368 |

| | | | | | | | |
|---|---|---|---|---|---|---|---|
| | | | | 4 | 0.072 | 0.204 | 1.833 |
| | | | | 5 | 0.069 | 0.409 | 4.928 |
| 0.05 mg OVA | IP 28-days | 5 | 0 and 35 | 1 | 0.085 | 1.256 | 13.7765 |
| | | | | 2 | 0.056 | 0.423 | 6.5536 |
| | | | | 3 | 0.073 | 2.255 | 29.8904 |
| | | | | 4 | 0.064 | 0.711 | 10.1094 |
| | | | | 5 | 0.072 | 1.675 | 22.2639 |
| | | | | 6 | 0.067 | 0.697 | 9.4030 |
| PBS | IP 28-days | 5 | 0 and 35 | 1 | 0.073 | 0.07 | -0.0411 |
| | | | | 2 | 0.07 | 0.072 | 0.0286 |
| | | | | 3 | 0.069 | 0.066 | -0.0435 |
| | | | | 4 | 0.074 | 0.077 | 0.0405 |
| | | | | 5 | 0.079 | 0.067 | -0.1519 |
| | | | | 6 | 0.075 | 0.069 | -0.0800 |
